# Targeting Recovery in Persistent Persecutory Delusions: A Proof of Principle Study of a New Translational Psychological Treatment (the Feeling Safe Programme)

**DOI:** 10.1017/S1352465816000060

**Published:** 2016-04-05

**Authors:** Daniel Freeman, Jonathan Bradley, Felicity Waite, Bryony Sheaves, Natalie DeWeever, Emilie Bourke, Josephine McInerney, Nicole Evans, Emma Černis, Rachel Lister, Philippa Garety, Graham Dunn

**Affiliations:** University of Oxford, UK; King's College London, Institute of Psychiatry, Psychology, and Neurosciences, UK; University of Manchester, UK

**Keywords:** Delusions, paranoia, cognitive, treatment

## Abstract

**Background:** Many patients do not respond adequately to current pharmacological or psychological treatments for psychosis. Persistent persecutory delusions are common in clinical services, and cause considerable patient distress and impairment. Our aim has been to build a new translational personalized treatment, with the potential for wide use, that leads to high rates of recovery in persistent persecutory delusions. We have been developing, and evaluating individually, brief modular interventions, each targeting a key causal factor identified from our cognitive model. These modules are now combined in “The Feeling Safe Programme”. **Aims:** To test the feasibility of a new translational modular treatment for persistent persecutory delusions and provide initial efficacy data. **Method:** 12 patients with persistent persecutory delusions in the context of non-affective psychosis were offered the 6-month Feeling Safe Programme. After assessment, patients chose from a personalized menu of treatment options. Four weekly baseline assessments were carried out, followed by monthly assessments. Recovery in the delusion was defined as conviction falling below 50% (greater doubt than certainty). **Results:** 11 patients completed the intervention. One patient withdrew before the first monthly assessment due to physical health problems. An average of 20 sessions (*SD* = 4.4) were received. Posttreatment, 7 out of 11 (64%) patients had recovery in their persistent delusions. Satisfaction ratings were high. **Conclusions:** The Feeling Safe Programme is feasible to use and was associated with large clinical benefits. To our knowledge this is the first treatment report focused on delusion recovery. The treatment will be tested in a randomized controlled trial.

## Introduction

Persecutory delusions, a central problem in schizophrenia, are unfounded beliefs that others are trying to harm the person (Freeman and Garety, 2000). Almost half of patients with persecutory delusions have major depression (Vorontsova, Garety and Freeman, 2013) and levels of psychological well-being in the lowest 2% of the population (Freeman, Startup, et al., 2014). The presence of persecutory delusions predicts suicide (Hor and Taylor, 2010), serious violence (Coid et al., 2013), and hospital admission (Castle, Phelan, Wessely and Murray, 1994). It is well-recognized that treatments for persecutory delusions need significant improvement. The first line treatment, antipsychotic medication, has effect sizes (standardized mean differences) varying between 0.33 and 0.88 (median=0.44) (Leucht et al., 2013), with problems of major side-effects, poor compliance, and residual problems. A meta-analysis for the effects on delusions of first generation cognitive-behavioural treatments (when added to medication) indicates an effect size of 0.36 (van der Gaag, Valmaggia and Smit, 2014), while there are significant problems of implementation in services for this approach (Haddock et al., 2014). Treatment effect sizes in psychosis are lower than those found for common mental health conditions such as anxiety disorders. Our aim has been to translate theoretical advances, guided by a cognitive model (Freeman, in press; Freeman, Garety, Kuipers, Fowler and Bebbington, 2002), into a much more efficacious and easily disseminated treatment that can lead to high recovery rates for persistent persecutory delusions.

### A cognitive perspective on persecutory delusions

We view persecutory delusions as the severe end of a paranoia continuum in the general population. Many people have a few paranoid thoughts, and a few have many (e.g. Bebbington et al., 2013). Key to our psychological conceptualization is the idea that at the core of a persecutory delusion is a threat belief (Freeman et al., 2002). The individual believes that he or she is currently unsafe. Genetic and environmental risk leads to such fears (Zavos et al., 2014). Once developed, the beliefs concerning danger are maintained by a number of key factors (Freeman and Garety, 2014; Freeman, in press): worry brings implausible ideas to mind, keeps them there, and exacerbates the distress; negative self-beliefs lead the person to feel inferior and vulnerable; subjectively anomalous internal states (e.g. dissociation, unexplained anxious arousal, hallucinations) provoke fearful explanations; disrupted sleep increases negative affect, mood dysregulation, and anomalous internal states; reasoning biases prevent the processing of alternative explanations; and safety-seeking behaviours such as avoidance prevent the person receiving and processing disconfirmatory evidence that they are safe. Therefore treatment needs to target the maintenance factors, before helping the patient to go into everyday situations and relearn that they are safe. Our psychological treatment goal is to establish a new belief concerning current safety. In this way, the threat belief recedes and recovery in the delusion occurs (defined in psychiatric terms as greater doubt than certainty in the delusion). This is a key clinical goal in itself: patients desire to feel less paranoid (Bryne and Morrison, 2014). But, importantly, other benefits inevitably accrue in the wake of reducing the threat belief, such as less paranoia-related distress, greater psychological well-being, and higher activity levels as re-engagement with the social world occurs.

### Developing a new treatment

In order to build a new treatment we have been developing brief intervention modules, highly manualized and delivered one-to-one with a therapist, that each target a key maintenance factor identified from our cognitive model. Five modules have now been separately evaluated in randomized controlled tests - varying from proof of concept experiments to pilot studies to efficacy trials - to show that each element merits inclusion in a full treatment. The methodologically strongest clinical test has been for reducing worry. A randomized controlled trial (The Worry Intervention Trial) with 150 patients with persistent persecutory delusions had blind ratings and a 95% follow-up rate (Freeman, Dunn, et al., 2015). Targeting worry, in just six sessions, significantly reduced both worry and the persecutory delusions (both effect sizes = 0.5). There were also improvements in every secondary outcome measure, including overall levels of psychiatric symptoms and psychological well-being. A pilot randomized controlled trial (The Self-Confidence Study), with 30 patients with persistent persecutory delusions, principally used techniques to enhance positive self-beliefs in order to limit the effects of negative self-beliefs (Freeman, Pugh, et al., 2014). Ratings were blind and 100% of patients were followed up. Posttreatment there were improvements in positive self-beliefs (effect size = 1.0) and psychological well-being (effect size = 1.2) and reductions in negative self-beliefs (effect size = 0.24) and the delusions (effect size = 0.6). An assessor-blind pilot randomized controlled trial (The Better Sleep Trial) with 50 patients with persistent delusions and hallucinations showed that sleep can be substantially improved (effect size = 1.9) and that there may be consequential benefits in levels of paranoia (effect size = 0.2) and quality of life (effect size = 0.5) (Freeman, Waite, Startup, et al., 2015). Two randomized controlled studies have also shown the value of focused work reducing reasoning biases in patients with delusions (Garety et al., 2015; Waller et al., 2015). In a pilot clinical study with 31 patients with persistent delusions, the Thinking Well reasoning intervention led to a reduction in delusional conviction (effect size = 0.6) compared to standard care (Waller et al., 2015). Finally, the most recent study shows that testing the predictions of the persecutory threat beliefs by entering feared situations while dropping safety behaviours reduces the delusions to a much greater extent than exposure alone (effect size = 1.3) (Freeman et al., in press).

### The current feasibility study

The treatment elements, each individually evaluated, have now been combined in the Feeling Safe Programme. Treatment is personalized and includes patient preference. We explain to patients that the goal of the programme is to help them to feel safer, happier, and be more active. At the end of the first session a menu of treatment options – based upon questionnaire assessments and a brief clinical interview – is provided for patients. Patients choose their preferred treatment elements and the order of implementation. Once a maintenance factor is reduced, therapy progresses to the next relevant cause. The full treatment avoids overly complex formulations, instead using clear personalized explanations that contain an encouraging rationale for how change can occur. Progress is monitored throughout and the patient is encouraged to learn through direct experience. In-person sessions are weekly, but frequent contact concerning the implementation and adjustment of change strategies is expected. In the current study we set-out to use the Feeling Safe Programme in a first cohort of patients. All patients were offered treatment. Principally we aimed to assess feasibility with regard to treatment up-take, identification of the techniques used, and patient satisfaction. We also aimed to provide a preliminary assessment of potential clinical benefits with regard to the delusions, overall levels of psychiatric symptoms, and psychological well-being. Hypothesized maintenance factors were assessed in order to inspect successful targeting of the mechanisms. It can be considered as a Phase I proof-of-principal study.

## Method

### Participants

Twelve patients were recruited from adult mental health teams in Oxford Health NHS Foundation Trust. The inclusion criteria were: a persistent persecutory delusion (which had lasted at least 6 months despite treatment) meeting the criteria of Freeman and Garety (2000) (an unfounded belief that harm is occurring, or is going to occur, to him or her and that the persecutor has the intention to cause harm); aged 18 to 70; a diagnosis of schizophrenia, schizo-affective disorder, delusional disorder, or psychosis NOS (i.e. non-affective psychosis).The exclusion criteria were: a primary diagnosis of alcohol or substance dependency or personality disorder; organic syndrome or learning disability; a command of spoken English inadequate for engaging in psychological therapy; current engagement in any other individual therapy. After completing the baseline assessments, one patient had a physical health problem that led to the stopping of medication, a substantial worsening of mental state, and a psychiatric hospital admission. Taking part in therapy or the assessments was not considered appropriate at this point. The patient did not complete any follow-up assessments and therefore this patient's data are not presented in the results section. Ethical approval was received from an NHS research ethics committee. The first patient began the study on 25 June 2014, and the last patient completed the final assessment on 16 July 2015.

### Design

The study had an A-B design. Patients were each in the study for a total of 8 months. There were four weekly baseline assessments with a research assistant before the treatment began. Following the baseline assessment there were seven monthly assessments carried out by a research assistant. Treatment was provided after the baseline assessments for up to 6 months.

### Assessments

Assessment of the delusions was made at all time-points using the Psychotic Symptoms Rating Scale-Delusions (PSYRATS) (Haddock, McCarron, Tarrier and Faragher, 1999). This included obtaining a 0–100% rating of the main persecutory delusion. Delusion recovery was defined as the conviction in the delusional belief falling below 50% i.e. that there was now greater doubt than belief in the delusion. Conviction greater than 50% is a standard definition of the presence of a delusion (e.g. Hartley, Haddock and Barrowclough, 2012), although typically such beliefs are held with much greater certainty. A PSYRATS delusion distress score was created by summing the distress amount and intensity items (e.g. Woodward et al., 2014). Overall levels of paranoia were assessed at every time-point using the Green et al. (2008) Paranoid Thoughts Scale (GPTS). At the first and last baseline assessments, and at all monthly assessments, overall levels of psychiatric symptoms were assessed with the Positive and Negative Syndrome Scale (PANSS) (Kay, 1991) and psychological well-being with the Warwick-Edinburgh Mental Well-Being Scale (WEMWBS) (Tennant et al., 2007). Patient satisfaction ratings were obtained at the end of therapy using an adapted 7-item version of the Client Satisfaction Questionnaire (Attkisson and Zwick, 1982). At the first and last baseline assessments, and at all the monthly assessments, we assessed potential mechanism variables using: the Penn State Worry Questionnaire (PSWQ; Meyer, Miller, Metzger and Borkovec, 1990); Brief Core Schema Scales (BCSS; Fowler et al., 2006); Depression Anxiety and Stress Scales – Depression and anxiety subscales (DASS; Lovibond and Lovibond, 1995); Insomnia Severity Index (ISI; Bastien, Vallieres and Morin, 2001); beads task (Garety et al., 2005); and belief flexibility (the possibility of being mistaken) (Waller et al., 2015). Levels of medication use throughout the trial were recorded.

### Adverse events

During the trial any adverse event that came to our attention was recorded. Medical notes were also checked at the end of the trial for the following events pre-specified as adverse: 1. All deaths. 2. Suicide attempts. 3. Serious violent incidents. 4. Admissions to forensic units. 5. Formal complaints about therapy.

### The intervention

Treatment was provided by clinical psychologists, using a consultant model for delivery. At the first treatment meeting, DF led an assessment with the patient, with one or two newly-qualified clinical psychologists from the team present (JB, BS, FW). The results of the questionnaire assessments were also used to inform the meeting. In the closing stages of this appointment, a menu of treatment options was offered by DF to the patient. Patient preference was used to select the first treatment target. Formulation was kept very simple at this stage (e.g. “It seems your sleep problems are making things worse?”, “Your self-confidence around other people is a problem?”, “Worry brings the worst ideas into your mind and makes you upset?”). Within each module the formulation was expanded for a specific causal factor. All interventions were framed positively (e.g. improving sleep, improving self-confidence, feeling safer). The first selected intervention was then provided by a clinical psychologist (JB, BS, FW), with occasional support for specific behavioural tasks from an assistant psychologist. The modules were delivered in a one-to-one format, with supportive telephone calls, texts, or e-mails between sessions. The therapy targets for each module were measured every session. Sessions were planned to last one hour, but allowing flexibility. At regular intervals, there would be a review attended by the patient, DF, and the other clinical psychologists. This would principally occur when a new module needed selection, and further clinical interviewing and the results of the monthly assessments informed this choice. Treatment was offered in approximately 20 sessions over 6 months (with gradual tapering). It was provided at the local mental health centre, or the patient's home, or environments in which learning could take place (e.g. the local shopping centre or park). The range of treatment options offered were: improving sleep, reducing worry, increasing self-confidence, improving reasoning processes, and learning that safer now than feared (real-life behavioural tests with dropping of safety behaviours). Throughout, re-engagement in positive activity was a means of implementing strategies. In the final sessions, a joint blue-print of therapy was written, including an action plan for if things became difficult again. The work was conducted, where possible, with close contact with the responsible clinical team. The therapists received weekly supervision from DF.

### Analysis

At this stage of treatment evaluation the focus was on feasibility and providing descriptive summary statistics for the outcome variables. Paired *t*-tests were conducted to assess change in the key outcomes. An average baseline score from the four baseline assessments was compared against the score at the patient's end of therapy assessment. The analysis did not include reporting *p* values, following recommendations that “The analysis of a pilot study should be mainly descriptive or should focus on confidence interval estimation.” (Lancaster, Dodd and Williamson, 2004). Effect sizes (Cohen's *d*) were calculated by dividing the change score obtained from the *t*-test by the standard deviation of the baseline average score. All statistical testing was conducted using SPSS Version 20.0 (IBM, 2011).

## Results

### Basic demographic and clinical information

Typical of studies of patients attending services with persistent psychotic experiences, the average age was 41.6 (*SD* = 15.2) years old, most patients were unemployed (*n* = 7) or part-time employed (*n* = 2), and there were more men (*n* = 6) than women (*n* = 5) taking part. The ethnicities were: White (*n* = 9), Asian (*n* = 1), mixed (*n* = 1). The clinical diagnoses were: schizophrenia (*n* = 4), schizo-affective disorder (*n* = 5), delusional disorder (*n* = 1), psychosis NOS (*n* = 1). Auditory hallucinations were highly prevalent (*n* = 8). All patients were taking psychiatric medication, and most were taking two types. All but one patient was taking antipsychotic medication. All but one patient was taking anti-depressant medication. Two patients were also prescribed anxiolytic medication, and one patient was also prescribed a hypnotic.

### Uptake of the Feeling Safe Programme

The average number of sessions received by the patients was 20.0 (*SD* = 4.4) (minimum = 9, maximum = 24). One patient completed the intervention in 3 months, two patients completed the intervention in 5 months, and eight patients completed the intervention in 6 months. The first modules undertaken were reducing worry (*n* = 5), improving sleep (*n* = 3), and increasing self-confidence (*n* = 2). One patient arrived at the first session experiencing panic attacks (triggered by a family issue), and tackling these using standard CBT techniques was the first clinical priority, before moving on to the improving sleep module. Patients most commonly completed three modules (*n* = 5) or two modules (*n* = 4), with one patient completing four modules and one patient completing one module. The modules used were: improving sleep (*n* = 9), reducing worry (*n* = 6), increasing self-confidence (*n* = 3), improving reasoning processes (*n* = 2), and learning that safer than feared (*n* = 8). Satisfaction with therapy scores are provided in Table 1, showing high levels of approval by most of the patients.

**Table 1.** Satisfaction with therapy

Questionnaire item	Scale rating				
How would you rate the quality of the therapy that you have received?	Very poor *n* = 0	Poor *n* =0	Fair *n* =1	Good *n* = 3	Excellent *n* =7
Did you get the kind of therapy that you wanted?	No, definitely not *n* = 0	No, not really *n* = 0	Somewhat *n* =4	Yes, generally *n* = 2	Yes, definitely *n* = 5
If a friend were in need of similar help, would you recommend the programme?	No, definitely not *n* = 0	No, probably not *n* = 0	Unsure *n* = 4	Yes, probably *n* = 1	Yes, definitely *n* = 6
How satisfied are you with the amount of therapy that you have received?	Quite dissatisfied *n* = 0	Mildly dissatisfied *n* = 0	Somewhat satisfied *n*= 1	Mostly satisfied *n* = 3	Very satisfied *n* = 7
Has the therapy helped you to deal more effectively with your problems?	No, it hasn't helped at all *n* = 0	No, it didn't really help *n* = 0	Unsure if it has helped *n* = 1	Yes, it has helped *n* = 4	Yes, it's helped a great deal *n* = 6
In an overall, general sense, how satisfied are you with the therapy you have received?	Quite dissatisfied *n* = 0	Mildly dissatisfied *n* = 0	Somewhat satisfied *n* = 1	Mostly satisfied *n* = 3	Very satisfied *n* = 7
If you were to seek help again, would you come back to our programme?	No, definitely not *n* = 0	No, I don't think so *n* = 0	Unsure *n* = 3	Yes, I think so *n*= 0	Yes, definitely *n* = 8

### Clinical outcomes

Tables 2 and 3 provide summary data for the main outcomes and the assessments of the potential maintenance factors. It can be seen that upon entering the trial, the delusions were severe, overall levels of psychiatric symptoms high, and psychological well-being very low. As treatment progresses, there is improvement in the delusions, overall symptoms, and psychological well-being. At the point that each patient completed therapy, 7 out of 11 (64%) patients had delusion recovery. (Three out of the four patients with schizophrenia showed this recovery, three out of the five patients with schizo-affective disorder, and one out of two of the other non-affective psychosis clinical diagnoses.) Delusional conviction clearly reduced from the baseline period to after intervention, mean change = −36.7, 95% confidence interval (C.I.) = −69.3, −4.1, *d* = 1.5. Similarly, there were very large reductions in total delusion severity, PSYRATS total mean change =-6.7, 95% C.I. = −12.1,−1.2, *d* = 2.3, and delusion distress, PSYRATS distress mean change = −2.4, 95% C.I. = −4.47,−0.4, *d* = 2.2. There was also evidence of potential benefits in overall levels of paranoia, GPTS mean change = −30.0, 95% C.I. = −63.4, 3.4, *d* = 1.1, overall levels of psychiatric symptoms, PANSS mean change = −13.0, 95% C.I. = −27.7, 1.7, *d* = 0.8, and psychological well-being, WEMWBS mean change = 8.4, 95% C.I. = 18.4,−1.7, *d* = 0.9. The mean scores in Table 3 also indicate improvement in all of the maintenance factors assessed, apart from jumping to conclusions.

**Table 2.** Summary scores for the main outcome measures at each assessment point

	Mean score (*SD*)											
		Delusion		Delusion		Delusion		Paranoia		Total		Well-
Assessment		conviction		total		distress		total		symptoms		being
point	*n*	(%)	*n*	(PSYRATS)	*n*	(PSYRATS)	*n*	(GPTS)	*n*	(PANSS)	n	(WEMWBS)
Baseline 1	11	86.2 (17.6)	11	18.4 (1.5)	11	7.0 (1.0)	11	118.6 (18.6)	11	80.1 (17.9)	11	32.6 (11.1)
Baseline 2	11	72.5 (30.5)	11	16.4 (3.5)	11	6.45 (0.9)	10	115.8 (33.1)				
Baseline 3	11	82.3 (23.6)	11	15.4 (3.5)	11	5.6 (1.9)	10	108.9 (31.1)				
Baseline 4	11	74.9 (37.2)	11	15.8 (5.1)	11	5.8 (2.0)	11	104.2 (37.6)	11	79.3 (17.3)	10	29.1 (12.5)
Month 1	11	65.9 (33.7)	11	14.3 (5.1)	11	5.6 (2.4)	11	105.8 (30.7)	11	72.6 (18.3)	11	34.8 (11.7)
Month 2	11	57.5 (41.8)	11	12.0 (5.6)	11	4.6 (2.2)	10	83.8 (35.4)	10	69.9 (17.0)	11	42.4 (12.9)
Month 3	11	52.3 (42.5)	11	11.6 (7.7)	11	4.4 (3.0)	11	90.3 (40.0)	11	71.2 (16.4)	10	38.6 (14.3)
Month 4	9	40.1 (40.9)	9	8.8 (7.5)	9	3.1 (3.0)	9	72.7 (31.3)	9	67.2 (16.5)	8	41.1 (13.3)
Month 5	10	37.9 (42.3)	10	9.5 (6.5)	10	3.7 (2.6)	9	81.2 (45.9)	10	65.2 (16.8)	10	37.5 (12.9)
Month 6	11	52.1 (44.9)	11	11.9 (7.4)	11	4.5 (3.0)	11	80.6 (42.5)	11	69.2 (18.5)	11	37.5 (13.8)
Month 7	10	35.9 (42.0)	10	10.1 (6.7)	10	4.2 (3.0)	10	79.5 (39.8)	10	61.7 (16.4)	10	40.7 (9.5)

**Table 3.** Summary scores for the mechanism variables at each assessment point

	Mean score (*SD*)											
Assessment		Worry		Negative		Negative		Insomnia		Data-gathering		Possibility
point	*n*	(PSWQ)	*n*	self (BCSS)	*n*	affect (DASS)	*n*	(ISI)	n	(JTC)	n	mistaken (%)
Baseline 1	11	67.4 (9.3)	11	13.1 ((7.9)	11	51.1 (21.9)	11	17.2 (7.4)	10	5.1 (5.9)	11	36.7 (31.6)
Baseline 4	11	64.7 (8.7)	11	13.1 (8.2)	11	50.2 (23.1)	11	13.0 (9.6)	11	5.6 (6.5)	11	30.4 (30.2)
Month 1	11	63.7 (9.8)	11	10.8 (7.0)	11	48.6 (22.7)	11	16.2 (6.5)	11	6.3 (6.0)	11	48.1 (31.4)
Month 2	10	56.7 (15.4)	10	9.7 (7.8)	11	31.6 (22.9)	10	13.8 (9.3)	10	3.6 (3.7)	11	48.2 (35.0)
Month 3	11	57.8 (13.5)	11	8.2 (7.8)	11	36.9 (26.7)	10	14.4 (6.5)	11	5.5 (6.4)	11	56.7 (41.1)
Month 4	9	52.4 (17.5)	9	7.0 (7.1)	9	32.1 (18.7)	9	11.0 (7.0)	9	4.9 (3.9)	9	60.7 (40.4)
Month 5	10	54.2 (19.6)	10	7.5 (9.0)	10	31.8 (24.6)	10	10.8 (6.9)	10	4.3 (6.3)	10	57.4 (39.7)
Month 6	11	50.6 (13.9)	11	9.2 (9.6)	11	31.6 (22.4)	11	13.2 (7.9)	10	4.3 (6.2)	11	62.8 (40.0)
Month 7	9	53.2 (17.1)	10	6.9 (8.1)	10	27.3 (19.3)	10	10.2 (7.3)	9	5.0 (6.5)	9	52.9 (35.5)

### Medication, hospital admissions, and adverse events

There was a gradual reduction in levels of medication prescribed. At the start the chlorpromazine equivalent dose was 372.7 (*SD* = 247.1) mg/day (*n* = 11), while at 6 months it was 239.4 (*SD* = 210.7) (*n* = 11). By the end of the trial three patients were no longer being prescribed antipsychotic medication. One of the eleven patients (who was maintained on medication) had an admission. No adverse events occurred for the trial participants during their 8 months in the study.

## Discussion

Patients with persistent persecutory delusions, often held for many years despite standard psychiatric care, took part in this first evaluation of a new translational treatment. The treatment was very well-received by patients. Up-take of the Feeling Safe Programme was high. The amount and quality of the therapy was judged by the patients as appropriate; almost all of the patients believed that the treatment had helped them to deal more effectively with their problems; and the majority of trial patients were clearly highly satisfied with the Feeling Safe Programme. Improvements assessed by the clinical measures were in the large effect size range. The majority of patients, though not all, achieved recovery in their persecutory delusions. These are striking effects and in contrast to a view that this is a group of patients that inevitably has problems of compliance with treatment and long-standing intractable difficulties. In essence, the modular interventions first aimed to stabilize circadian rhythms, reduce negative affective states, and increase activity. (In a small number of cases, directly reviewing reasoning processes which maintain paranoid beliefs was also conducted.)Trust in the therapists gained from such progress was then used to encourage patients to go on to the direct work of building up multiple experiences that they were safer than they had feared. Building up a belief about safety was used to counteract the threat belief at the heart of persecutory delusions.

From this experience there are several elements of the style of the intervention that we consider important. The formulation messages have been distilled. It was explicitly communicated that the origin of problems is complex, but that the key factors are best tackled one at a time. There is comparatively little time spent going over the past, although many of the patients, had had difficult life experiences that needed appropriate acknowledgement in their contribution to current problems. Instead the focus is on the present and future. Generally there is no value in evaluating whether the delusion was accurate, but instead we examine its current veracity. Throughout, the therapy is deliberately active, often with sessions taking place in real-life situations in which learning can take place. There is an emphasis placed on the therapist- by the repeated measurement and the early implementation of change strategies - to demonstrate the value of the work. Too often this patient group has become dispirited by ineffective attempts at help. And we believe the consultant model worked well, consistent with the high retention rate of the study. Perhaps in contrast to what many clinicians might expect, the patients were able to be comfortable with several team members in the room. The reason – so that the whole team could work on the patient's behalf without the need for him or her to keep repeating information – was readily understood. This model also embeds close supervision and aids training, which is helpful in meeting the challenge of implementation.

In putting the modules together for the first time, we noted areas in which the intervention could be developed further. An obvious spur are the two clear instances where the treatment did not work. A common feature was the failure to get to the stage of direct learning of safety, although the reasons for this differed. One patient made it clear that the only direct delusion-related strategy that might be tried was altering the reactions to the perceived persecution (changing from upset, rumination, and anger to learning to ignore and focus upon positive activity). This type of work requires further manualisation in the programme. In the other case, a young patient with psychosis was only now sharing their severe psychotic experiences for the first time. The focus of therapy was on helping this sharing to occur, highlighting the different relationships that can be held with voices, and, most importantly, providing support during his preparing and taking of college exams (which was considered the priority). Guidance concerning hearing voices is provided within a number of the modules, but a stand-alone section on this topic and related anomalous experiences is an element we plan to add. The use of a modular approach in the Feeling Safe Programme is helpful in its flexibility, allowing for the later addition of other theoretically underpinned approaches that may emerge as clinically useful (e.g. Hayward, Strauss and Bogen-Johnston, 2014; van den Berg et al., 2015; Lincoln, Hartmann, Kӧther and Moritz, 2015; Morrison et al., 2014). The baseline scores of a number of the trial patients reduced slightly over time – one interpretation is that the delusions were already beginning to recover, but our view, given the previous persistence of the delusions, is that this reflects the early stages of change triggered by the frequent and relevant assessments. Further strengthening of the level of monitoring within the Feeling Safe Programme itself may be warranted. It is also of note that there was a reduction in the use of antipsychotic medication. As their delusions were recovering, several patients expressed their desire to not be on medication for the long-term. Close working with other mental health colleagues is needed and their doses were tapered in consultation with their psychiatrists.

The development of a treatment progresses through several stages over a number of years. This study was the first evaluation of the Feeling Safe Programme. A proof of principle study clearly has considerable limitations in what can be learned. In an uncontrolled study gains cannot be attributed with confidence to the intervention, even though a patient group was chosen who had not responded adequately to standard care. The assessments were not blind, which will introduce bias. The longer term effects were not assessed. It is also clear that full recovery from psychosis concerns more domains than only reductions in delusions (Law and Morrison, 2014). Nonetheless, we believe that the Feeling Safe Programme has considerable promise, given its theoretical bedrock, support from the trials testing the individual modules, and the current study's feasibility and efficacy results. It may well be a suitable treatment for emerging national schemes to improve access to psychological therapies for patients with psychosis (e.g. Jolley et al., 2015). We are now comparing this treatment in a randomized controlled test (The Feeling Safe Study) against an attention control condition (ISRCTN18705064) (Freeman, Waite, Emsley et al., 2016). Overall, we believe that the aim of targeting recovery in delusions is a key direction in the future of treatments for patients with psychosis.
